# *Rhodotorula taiwanensis* MD1149 produces hypoacetylated PEFA compounds with increased surface activity compared to *Rhodotorula babjevae* MD1169

**DOI:** 10.1371/journal.pone.0190373

**Published:** 2018-01-02

**Authors:** Mathew Lyman, Bonnee Rubinfeld, Roald Leif, Heather Mulcahy, Lawrence Dugan, Brian Souza

**Affiliations:** 1 Biosciences and Biotechnology Division, Lawrence Livermore National Laboratory, Livermore, California, United States of America; 2 Forensic Science Center, Lawrence Livermore National Laboratory, Livermore, CA, United States of America; University of Huddersfield, UNITED KINGDOM

## Abstract

Biosurfactants have several desirable characteristics in the industrial sector: detergency, antimicrobial effects, skin hydration, and emulsibility. Several yeast glycolipids are currently being utilized in these capacities: sophorolipids, ustilagic acid, and mannosylerythritol lipids (MELs). An emerging class of glycolipids, termed polyol esters of fatty acids (PEFA), have recently been reported for *Rhodotorula babjevae*, a basidiomycetous yeast species that secretes hyperacetylated congeners of PEFA (typically with 3–6 acetylation modifications). While screening Rhodotorula species for surfactant production, we identified a new environmental isolate identified as *Rhodotorula taiwanensis* MD1149 that dropped the surface tension of the liquid medium, indicating that it produced a potent biosurfactant. Acid depolymerization of the purified biosurfactants, followed by gas chromatography-mass spectrometry (GC-MS) analysis revealed that the biosurfactants were composed of PEFA compounds composed mainly of mannitol and arabitol esters of 3-hydroxy fatty acid, 3-methoxy fatty acid, and fatty acids with a single double bond; chain lengths were mainly C16 and C18. Liquid chromatography-mass spectrometry (LC-MS) confirmed the predicted accurate mass of these compounds. Interestingly, PEFA compounds produced by *Rhodotorula taiwanensis* MD1149 were more surface active due to their hypoacetylation profile (0–4 acetylation modifications) compared to *Rhodotorula babjevae* MD1169. These disparate surface active properties, based on acetylation, change the hydrophilic-lipophilic balance (HLB) of these compounds, and their potential utility within industrial applications.

## Introduction

Biosurfactants are small, amphiphilic molecules produced by a variety of microorganisms, namely bacteria, yeast, and fungi [1, 2]. They have broad applications for industrial usage, including the petroleum, cosmetics, pharmaceutical, detergents, paint, and food markets [3, 4]. Their demand continues to grow, with the global biosurfactant industry expected to exceed $2.2 billion USD by 2018 [5–7]. It is noteworthy that most industrial patents are held on four biosurfactant species: surfactin, rhamnolipids, sophorolipids, and mannosylerythritol lipids (produced by *Bacillus*, *Pseudomonas*, *Starmerella bombicola* (reclassified from *Candida bombicola)*, and *Pseudozyma* species, respectively) [8, 9]. Although these biosurfactants have shown utility in specific applications, there is a need to identify new classes of compounds that fill gaps within the biosurfactant HLB scale, thereby opening new avenues of biosurfactant application within the industrial complex[3, 8].

Biosurfactants have also gained popularity due to their “green factor”, i.e. their ability to be biodegradable—metabolized naturally by organisms in the environment—and biocompatible—less toxic to the ecosystem, especially in marine environments [4]. These compounds can be produced in a sustainable, eco-friendly manner that is less dependent on petroleum resources [10].

In the past six months, there has emerged an intense interest in developing polyol esters of fatty acids (PEFA) as industrial biosurfactants [11, 12]. These unique glycolipids are produced mainly by *Rhodotorula* yeast species, and are structurally distinct from other yeast glycolipids such as sophorolipids, ustilagic acid, and mannosylerythritol lipids (MELs). Garay et al. noted that PEFA compounds “may aid in the improving production of renewable, sustainable, environmentally friendly surfactants for use in household and industrial cleaning products, as well as many other applications [12].” In the process of screening *Rhodotorula* species for surfactant production, we recently identified a new strain, *Rhodotorula taiwanensis* MD1149 that produces hypoacetylated PEFA compounds compared to those recently published in the literature. This study describes the identification, characterization, and potential impact of these hypoacetylated PEFA compounds in this emerging field.

## Materials and methods

### Culturing of *Rhodotorula* strains

*Rhodotorula bogoriensis* was obtained from the American Type Culture Collection (ATCC 18809); note that the taxonomy of this strain was revised in 2015, and is listed in some collections under the current name *Pseudohyphozyma bogoriensis* [13]. *Rhodotorula taiwanensis* (MD1149) was obtained through M.J. Daly at the Uniformed Services University of the Health Sciences (USUHS); *Rhodotorula babjevae* (EXF-513/MD1169) was acquired via M.J. Daly through the Ex Culture Collection of Extremophilic Fungi, a part of the Infrastructural Centre Mycosmo (MRICUL) at the Department of Biology, University of Ljubljana, Slovenia. All yeast strains were grown in yeast mold broth (YM, Difco # 271120) overnight at 25°C and diluted to 0.05 OD_600_/mL in Hommel's minimal salts (HMS, per liter– 3 g (NH_4_)_2_SO_4_, 0.5 g NaCl, 0.7 g MgSO_4_, 0.4 g Ca(NO_3_)_2_, 0.4 g K_2_HPO_4_, 2.5 g KH_2_PO_4_) supplemented with 0.6 g/L yeast extract (Difco # 210929) and 50g/L glucose. At the indicated time (2, 4, 6 and 8 days), OD_600_ was assessed and 10 mL of culture were centrifuged twice at 5000*g to obtain spent liquid medium (SLM) for assays.

### Surface tension measurements (Tensiometry)

The surface tension was measured by the Wilhelmy plate method [14]. This method utilized a roughened platinum plate at room temperature coupled to a Kruss K11 force tensiometer. The Kruss measurement parameters used were as default with the exception of the following: Max measure time– 2000 s; # of values—200 and standard deviation—0.1 mN/m.

### Solid phase extraction

Biosurfactant compounds were purified from spent liquid medium using a 60 mL (10 g) Discovery C18 solid phase extraction tube (Supelco). The sorbent was conditioned with 50 mL of LC-MS grade methanol (Burdick and Jackson), followed by 50 mL of LC-MS grade water (Burdick and Jackson). Spend liquid medium was then added to the column (~40 mL), and allowed to flow through the column using gravity filtration (no vacuum). The column was then washed with an equal volume of water, followed by equal volumes of 20%, 40%, 60%, 80% and 100% methanol. Starting material, wash, and all eluates were then analyzed by LC-MS to determine in what fraction the compounds of interest eluted. The fraction of interest was then evaporated to dryness using a Savant SPD111V speedvac concentrator (Thermo Scientific) in preparation for composition analysis.

### Glycosyl composition and fatty acid analysis

Biosurfactant composition analyses were performed by gas chromatography-mass spectrometry (GC-MS) of the trimethylsilyl (TMS) derivatives of the polyols and the fatty acid methyl esters produced by methanolysis [15–17]. Briefly, the samples (200–300 μg) were placed in 4 mL vials with 0.5 mL of 1N methanolic HCl, sealed with a Teflon-lined screw caps, and placed in a heating block for 18 hours at 80°C. After cooling, the solvent was removed by nitrogen blowdown in a heating block set at 40°C. Next, the dried residues were treated with 0.5 mL Sylon HTP silylating agent (3:1:9 HMDS:TMCS:Pyridine) for 30 minutes at 80°C. After silylation, the excess derivatizing solution was removed by nitrogen blowdown in a heating block set at 40°C. The products were re-dissolved in 1 mL hexane. GC-MS analyses were performed on an Agilent 7890A gas chromatograph equipped with a 30 m x 0.25 mm i.d. HP-5ms capillary column (0.25 um film thickness) coupled to an Agilent 5975c mass spectrometer operated at 70 eV over the scan range m/z 29–600. The GC was heated using the following program: isothermal for at 40°C for 3 min., 8°C/min to 300°C and isothermal for 3.5 min., with the injector temperature set at 250°C. Flow rate of the helium carrier gas was set at a constant 1 mL/min. Compounds were verified with authentic standards. Fatty acid standards were obtained from Larodan Fine Chemicals.

### High resolution liquid chromatography-electrospray ionization-mass spectrometry (LC-ESI-MS)

Biosurfactants were detected in aqueous medium using an Agilent 6550 Accurate-Mass TOF LC-MS system. A reversed-phase Zorbax Eclipse Plus C18 (RRHD) column (2.1 x 100 mm, and 1.8 μm particle size) was used at 30°C in an Agilent 1290 HPLC Separation Module connected to an Agilent 6550 iFunnel Q-TOF LC-MS system equipped with an Agilent Jet Stream II dual sprayer ESI source. Mobile phases consisted of water-formic acid (99.9%:0.1%) (solvent A) and 100% acetonitrile (solvent B). The following solvent composition program was used: isocratic 0.5 min of 20% of solvent B, gradient for 19.5 min until 95% solvent B, isocratic 10 min with 95% solvent B, then an equilibration time of 5 min (post time). The flow rate was kept constant at 0.3 mL/min, the injection volume was 10 μl (with needle wash), and the samples were maintained at 4°C inside the autosampler. The LC-MS instrument was operated in positive ion electrospray mode with an acquisition range of 115–1700 amu with a scan rate of 3 spectra/sec. The source was kept at 225°C with a gas flow of 17 l/min, and a sheath gas temperature of 380°C and sheath gas flow of 12 l/min. The VCap was set at 3500, the nozzle voltage at 500 V, the fragmentor at 150, Skimmer1 to 0, and octopole RF peak to 750. Data acquisition was performed using Agilent MassHunter LC-MS Data Acquisition Software (version B.05.01, build 5.01.5125.2). Data analysis was performed using Agilent MassHunter Qualitative Analysis Software (version B.06.00, build 6.0.633.0).

## Results and discussion

*Rhodotorula bogoriensis*, recently reclassified as *Pseudohyphozyma bogoriensis* [13], was the first *Rhodotorula* species described as a sophorolipid producer [18]. It has continued to be a well-studied organism for biosurfactant production under different carbon and nitrogen sources [19, 20]. Utilizing *R*. *bogoriensis* as our biosurfactant-producing control strain, we began to systematically screen other *Rhodotorula* isolates for “novel” biosurfactant production, i.e. biosurfactant compounds that were markedly different from those produced by *R*. *bogoriensis*. While screening multiple strains for reduced surface tension in the growth medium, we identified *R*. *taiwanensis* as a potential candidate for new biosurfactant production.

**Fig 1:**
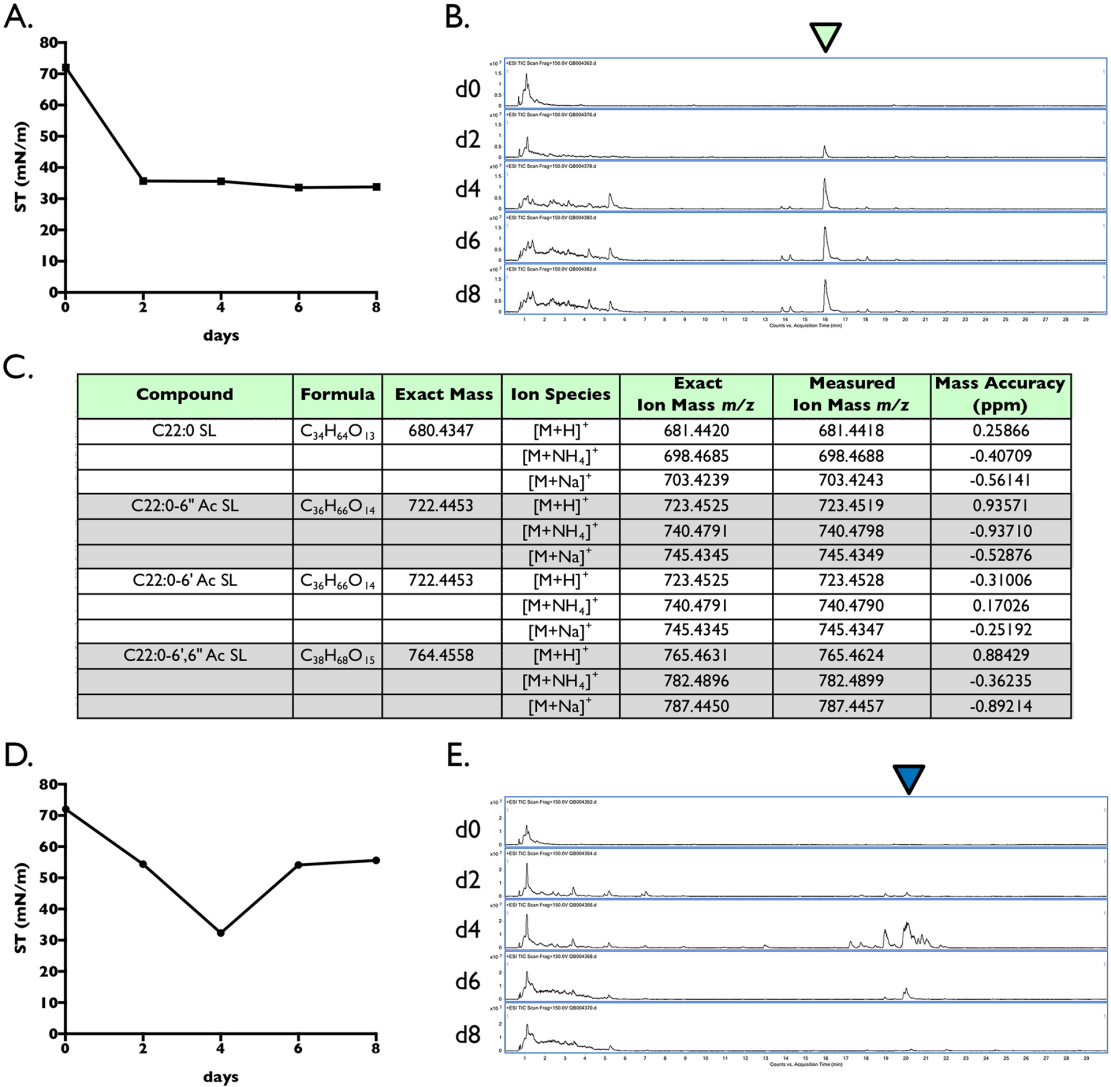
Biosurfactant production of *R*. *bogoriensis* (control) compared to *R*. *taiwanensis* MD1149. *R*. *bogoriensis* produced known sophorolipids that markedly reduced the surface tension of the culture medium (A). The presence of these sophorolipid biosurfactants was readily detected in the LC-MS total ion chromatograms (B, green triangle), and the accurate mass of the four main sophorolipid species was measured and confirmed (C). *R*. *taiwanensis* MD1149 produced biosurfactants that transiently lowered the surface tension of the culture medium (D), which corresponded with their appearance, and subsequent disappearance, in the LC-MS total ion chromatograms (E, blue triangle).

To confirm this, *R*. *bogoriensis* and *R*. *taiwanensis* strains were grown side-by-side for eight days in a minimal glucose medium supplemented with yeast extract, and measured every two days for dramatic drops in surface tension. In addition, samples were subjected to accurate mass LC-MS analysis to confirm the presence of a biosurfactant in the medium if a decrease in surface tension was observed. As shown in Fig 1A, we noted an immediate drop of surface tension in the medium of *R*. *bogoriensis* at day two, which stayed low for the duration of the time-course (ending at 33.8 mN/m). It is noteworthy that microbes that produce biosurfactants typically lower the surface tension of the growth medium from ~70 mN/m down to 25–35 mN/m [1]. LC-MS analysis revealed the presence of biosurfactants in the medium which were denoted by the green triangle above the total ion chromatograms in Fig 1B; these amphiphilic compounds elute later in the chromatographic gradient due to their fatty acid chains binding to the C18 column (thereby needing a higher % of organic solvent to elute), and are well separated from the more polar components in the growth medium that elute early in the LC-MS run.

High-resolution mass spectrometry measured the accurate mass of these *R*. *bogoriensis* compounds. An accurate mass measurement (“measured ion mass”), when compared to the calculated mass of an ion based on its elemental formula (“exact ion mass”), provides input for the mass accuracy calculation ∆*m*_*i*_ = (*m*_*i*_-*m*_*a*_)/m_*a*_ x 10^6^ in parts per million(ppm) where *m*_*i*_ is the measured ion mass and m_*a*_ is the exact ion mass. Mass accuracy subsequently determines the theoretical number of elemental formula that could match a particular ion species (reviewed in [21]). It is noteworthy that our mass accuracy for compounds measured was <1 ppm (Fig 1C), and matched only one elemental formula with the elements C, H, N, and O. These ion masses and formulae corresponded with the published masses of four sophorolipid species previously described for *R*. *bogoriensis* [19, 20]: deacetylated (C22:0 SL), monoacetylated (C22:0–6”Ac SL, C22:0–6’Ac SL), and diacetylated (C22:0–6’, 6” Ac SL).

By comparison, the surface tension profile of *R*. *taiwanensis* was markedly different; the surface tension dropped steadily down to ~32 mN/m over 4 days (log phase), then rose dramatically by day 6 (stationary phase), suggesting that the surface-active compounds were only transiently present in the culture (Fig 1D). LC-MS analysis confirmed this finding, demonstrating that biosurfactants in the growth medium peaked in concentration at day 4, and then quickly decreased by day 6 (denoted by the blue triangle above the total ion chromatograms in Fig 1E). We also noted that the surface-active compounds produced by *R*. *taiwanensis* were more hydrophobic than the sophorolipids produced by *R*. *bogoriensis* as observed by the longer retention time on the C18 column. The masses of the biosurfactants produced by *R*. *taiwanensis* compounds were also notably different than those reported for the sophorolipids produced by *R*. *bogoriensis*, and did not match any published masses for known biosurfactants. Therefore, we continued with our characterization.

**Fig 2:**
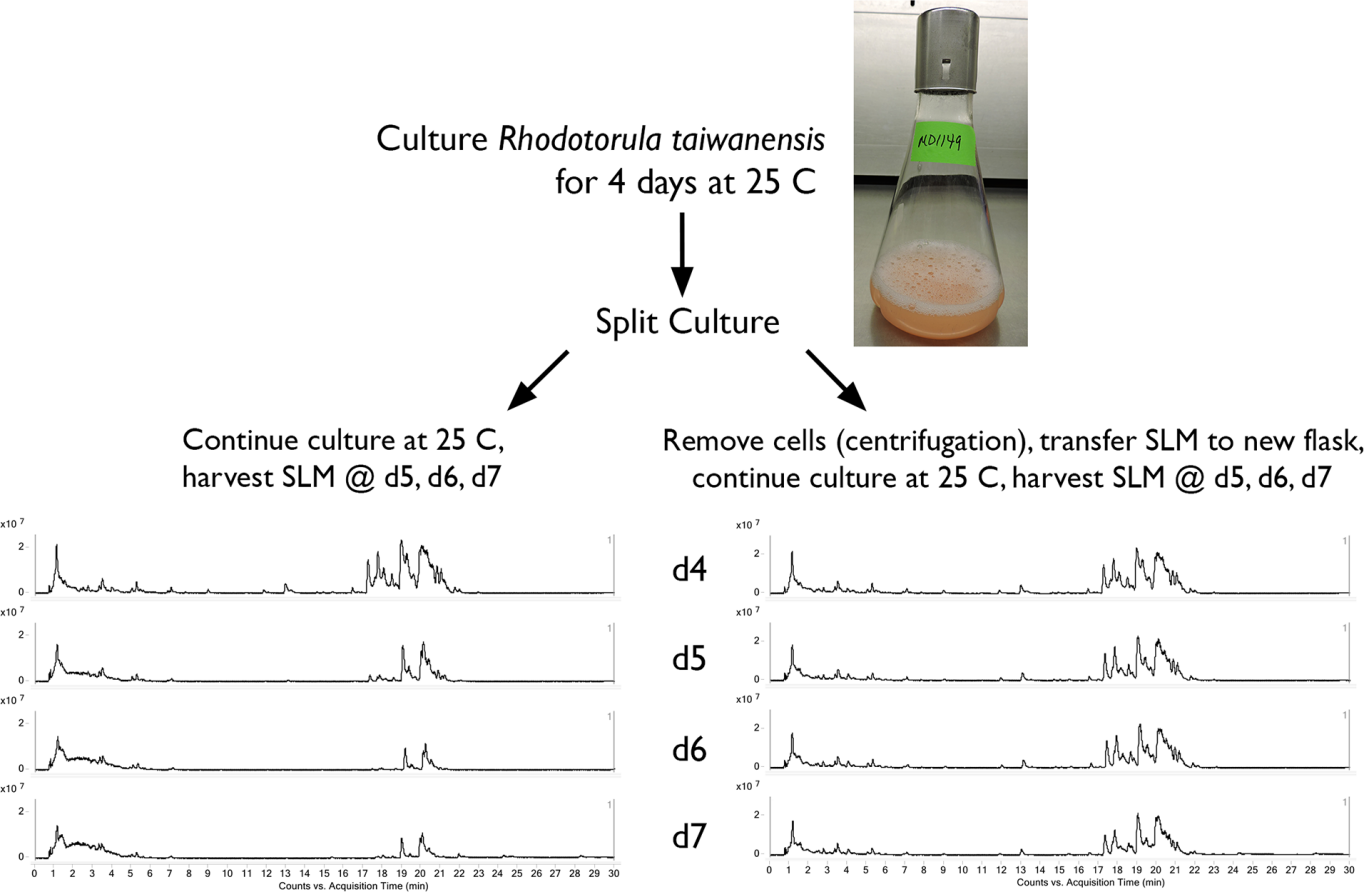
Biosurfactants produced by *R*. *taiwanensis* MD1149 are transiently present in the culture medium. MD1149 was cultured for four days (d4) then split; half of the culture was allowed to continue shaking with cells (left chromatograms), while in the other half, cells were removed via centrifugation, and allowed to continue shaking (right chromatograms). The two flasks were monitored for an additional three days (d5, d6, d7) by LC-MS.

Based on the appearance—and subsequent disappearance—of the biosurfactants in the culture medium (Fig 1E), we hypothesized that these compounds were being degraded directly by *R*. *taiwanensis* MD1149, and did not breakdown due to an inherent instability of the compounds in an aqueous environment. To test this idea, we prepared a shaking culture of *R*. *taiwanensis* as previously described. During maximum biosurfactant production (day 4), we split the culture as illustrated in Fig 2. Half of the culture was allowed to continue shaking at 25°C including cells; the other half of the culture was briefly centrifuged to remove cells, and the spent medium alone was transferred to a new flask and continued to shake at 25°C. A small volume of SLM was removed at days 5, 6, and 7 for LCMS analysis and side-by-side comparison for biosurfactant concentration. For the culture that was allowed to continue with cells, we observed a similar pattern as before; biosurfactant concentration was reduced to 34% by day 5, and 14% by days 6 and 7 (Fig 2, left chromatograms). By comparison, the culture that had cells removed maintained a steady concentration of biosurfactant in the SLM (Fig 2, right chromatograms). These findings suggest that the biosurfactant produced by *R*. *taiwanensis* MD1149 may be biodegradable, i.e. it may be metabolized by this yeast strain after being secreted into medium.

**Fig 3:**
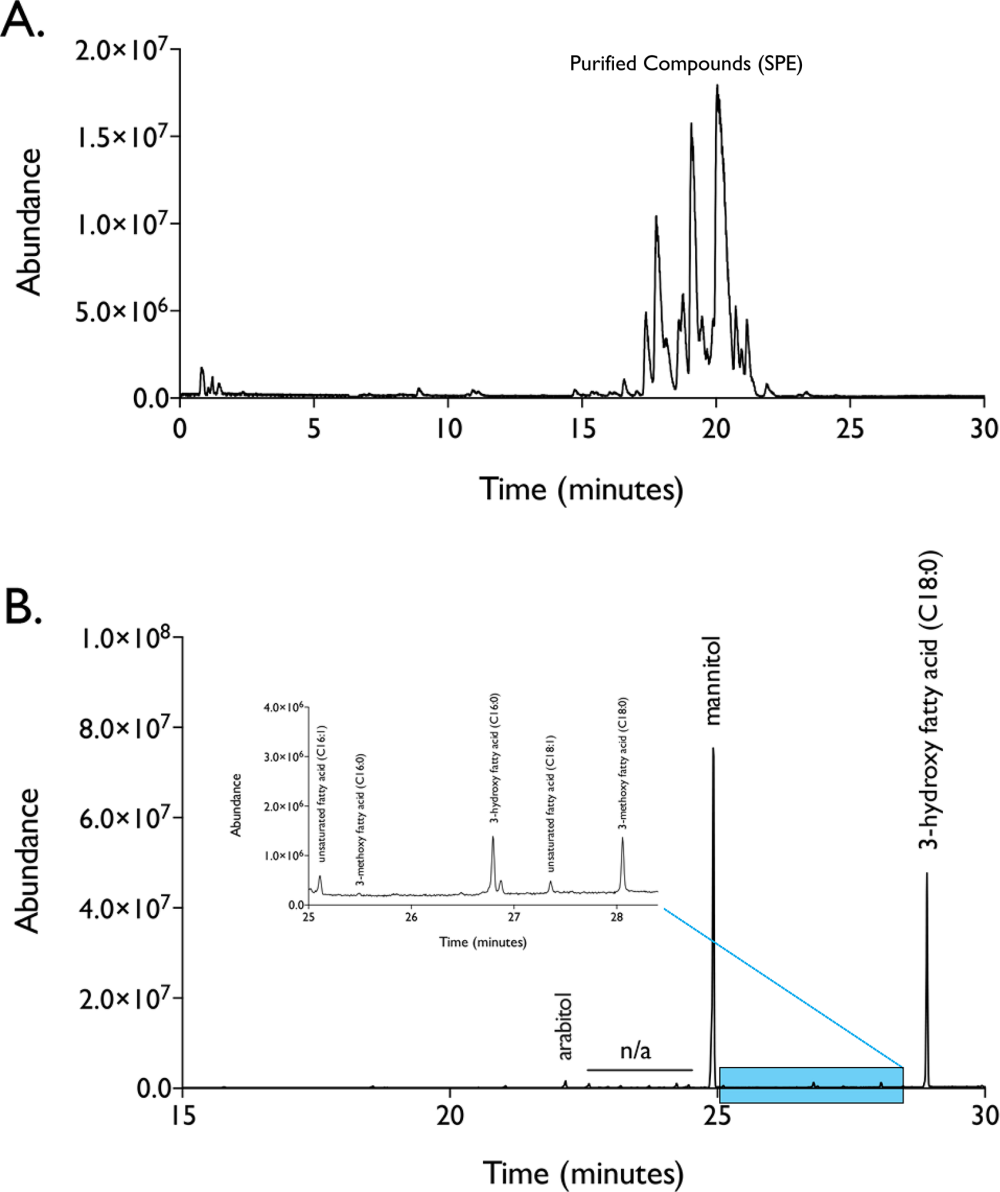
Biosurfactants produced by *R*. *taiwanensis* MD1149 are polyol esters of fatty acids (PEFA). Biosurfactant compounds were purified using solid phase extraction (reversed phase), and eluted from the column using 100% methanol as detected by LC-MS analysis (A). The organic solvent was subsequently evaporated, and the dried material was digested with methanolic HCl, derivatized (silylated), and analyzed by gas chromatography-mass spectrometry (B). GC-MS analysis revealed that the biosurfactant mixture was composed of glycolipids containing the sugar alcohols mannitol and arabitol (TMS derivatives), as well six main fatty acid constituents.

In order to better characterize the composition and structure of these compounds, we purified these biosurfactants from the culture medium using solid phase extraction (SPE). SPE is a sample preparation method that passes a liquid sample over a “bed” of solid particles that are normally packed into a column. Depending on the type of particles used, compounds of interest will bind to the column while other interferences/contaminants pass through the column and are removed; compounds of interest can then be eluted from the solid phase particles using relevant solvents. Given the hydrophobic nature of our compounds, we used a C18 SPE column (reversed-phase) that is well known to bind hydrophobic compounds; we then gently washed and eluted compounds from the column using an increasing amount of organic solvent (methanol). Polar and partially-polar compounds were eluted from the column using 20%, 40%, 60% methanol. Our compounds of interest begin eluting from the column at 80% methanol, and were fully removed using 100% methanol as examined by LC-MS analysis (Fig 3A).

It has been reported that yeast species can produce a wide variety of extracellular glycolipids such as sophorolipids, ustilagic acid, and mannosylerythritol lipids (reviewed in [22]; therefore, we hypothesized that the compounds produced by *R*. *taiwanensis* were also a type of fatty acid glycoside. To test this idea, we evaporated the solvent from the 100% eluate, and the dried material was digested, derivatized (silylated), and analyzed by gas chromatography–mass spectrometry (GC-MS). This method was used to determine the composition of glycolipids by separating the carbohydrate and fatty acid moieties through acid hydrolysis of the ester linkage(s) [1]. Subsequent silylation increased the volatility of the sugar moiety by replacing the hydrogen of the–OH groups with trimethylsilyl [23]. Derivatized sugars and fatty acid methyl esters (or fatty acids) could then be analyzed side-by-side in the same GC-MS run. Our analysis revealed that *R*. *taiwanensis* produced glycolipids composed of the sugar alcohols mannitol and arabitol (TMS derivatives), as well six main fatty acid constituents: 3-hydroxy stearic acid (C18:0), 3-hydroxypalmitic acid (C16:0), 3-methoxystearic acid (C18:0), 3-methoxypalmitic acid (C16:0), octadecenoic acid (C18:1, double bond in 2 position), and hexadecenoic acid (C16:1, double bond in 2 position). The relative abundances of these constituents are shown in Fig 3B, with mannitol and 3-hydroxy stearic acid (C18:0) being more abundant in the mixture (note: the mass spectra and retention times of these compounds were confirmed through a comparison with authentic standards). The relative abundance of the sugar alcohols to the fatty acids also showed that the they are in a 1:1 ratio, suggesting biosurfactants produced by *R*. *taiwanensis* are a mixture of polyol fatty acid esters; these compounds are similar in composition to extracellular glycolipids originally reported by Tulloch and Spencer [24], and more recently by Cajka et al [11].

**Fig 4:**
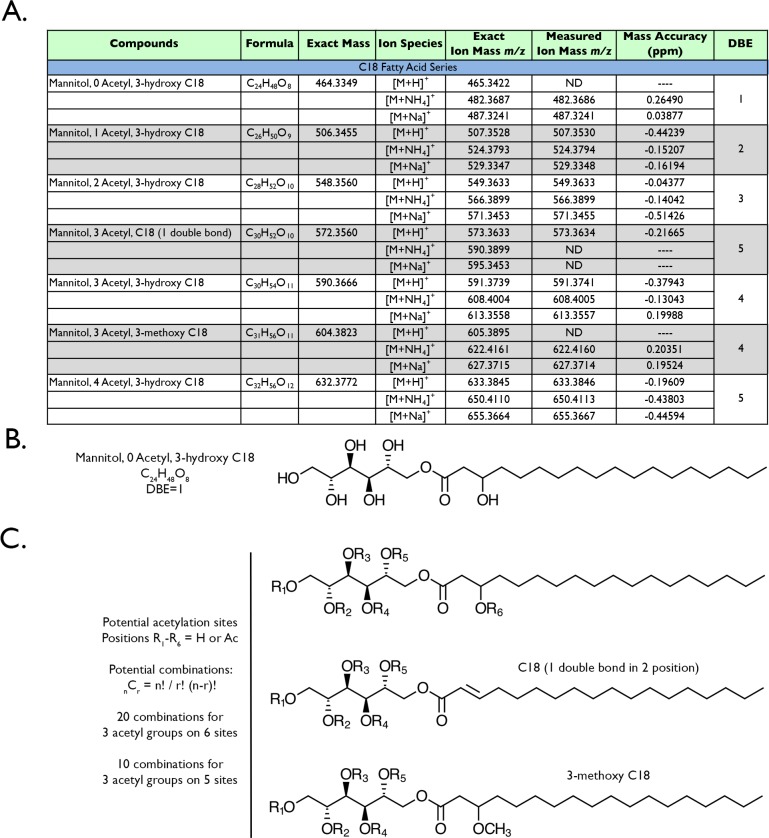
Mannitol 3-hydroxy C18 exists as an acetylation series. High-resolution mass spectrometry confirmed non-acetylated and acetylated mannitol 3-hydroxy C18 congeners in the spent liquid medium (A). The 3-methoxy and unsaturated fatty acid versions of these compounds were also detected. It is noteworthy that the calculated formulae also match the double bond equivalents (DBE) for the proposed structures (B and C). The potential acetylation sites (“R”) are highlighted on the different mannitol C18 congeners (C), as well as the potential number of structural combinations that exist for 3 acetyl groups—the most abundant type of mannitol 3-hydroxy C18.

Therefore, based on the GC-MS composition data, we began to build theoretical molecular formulae and structures of these compounds based on their constituent components. As we had already analyzed the SLM of *R*. *taiwanensis* cultures (n = 3) by high-resolution liquid chromatography-electrospray ionization-mass spectrometry (LC-ESI-MS), we began a targeted analysis of the accurate mass data using MassHunter Software with our calculated molecular formula. It is noteworthy that MassHunter uses a search algorithm that finds compounds via accurate mass compared to theoretical mass (of the formula), isotope abundance, and isotope spacing. We also searched for any one of three potential adducts of our compounds ([M+H]^+^, [M+NH_4_]^+^, [M+Na]^+^ knowing that adduct formation can be highly variable depending on the structure of the compound, the ion milieu of the spent liquid medium, and the mobile phase modifiers. As shown in Fig 4A (first row), we readily identified mannitol connected to a 3-hydroxy C18 fatty acid by LC-MS given their abundance, with an accurate mass of <0.26 ppm (confirming that there is no other molecular formula that fits the measured ion mass). The structure of this compound is shown in Fig 4B, and fits the calculated double bond equivalent (DBE) value—a “degree of unsaturation” calculator often used by structural chemists—to predict the number of double bonds in a proposed structure from a molecular formula; the mannitol 3-hydroxy C18 is calculated to contain a single double bond based on its formula, and does (Fig 4B).

Our LC-MS data also showed a series of hypoacetylated mannitol 3-hydroxy C18 compounds (Fig 4A), which were not detected by GC-MS analysis (due to acid digestion of the compounds which would remove the acetyl groups). Acetylation is readily detected by high-resolution mass spectrometry when an acetyl group (CH_2_CO) replaces the hydrogen on a hydroxyl group (OH), resulting in the addition of 42.0106 amu to the mass of the compound. For example, mannitol 3-hydroxy C18 has six potential acetylation sites (Fig 4C). Interestingly, we only detected compounds that contained 0–4 acetyl groups, with mannitol containing 3 acetyl groups being the most abundant in the SLM (compounds are listed in Fig 4A). Furthermore, only mannitol (3 acetyl groups) with 3-methoxy C18 and C18 (one double bond) fatty acids were detected by LC-MS; structures are shown in Fig 4C. Using the factorial equation _n_C_r_ = n!/r!(n-r)!, with “n” representing the potential number of acetylation sites and “r” representing the number of acetyl groups, we determined the total number of 3 acetyl combinations on the three different polyol C18 fatty acid esters (Fig 4C), e.g. 20 different acetylation combinations exist for the mannitol 3-hydroxy C18 backbone. These combinations would have the exact same mass, but slightly different retention times. We did detect multiple retention times of the same mass for this compound species, supporting the notion that multiple acetylation combinations exist in the mixture. However, we cannot definitively state where those acetyl groups are positioned on the mannitol 3-hydroxy C18 compound.

**Fig 5:**
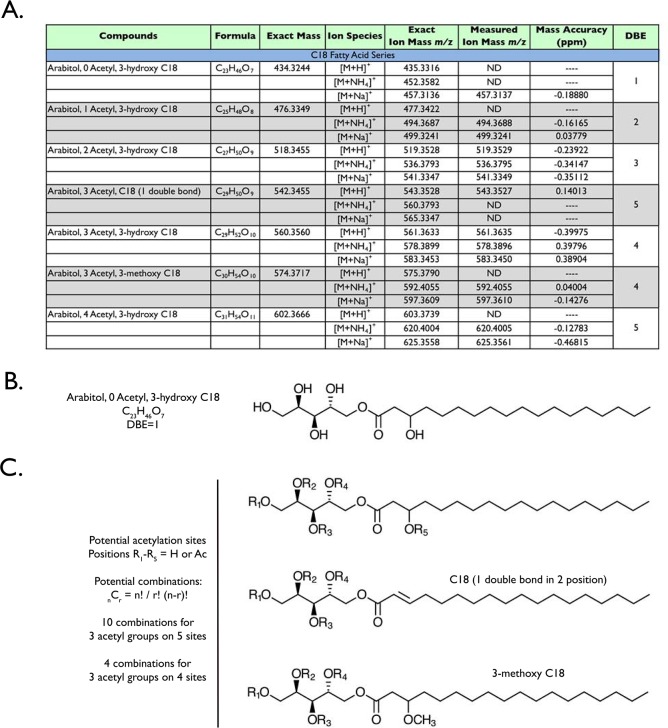
Arabitol 3-hydroxy C18 exists as an acetylation series. High-resolution mass spectrometry confirmed non-acetylated and acetylated arabitol 3-hydroxy C18 congeners in the spent liquid medium (A). The 3-methoxy and unsaturated fatty acid versions of these compounds were also detected. The calculated formulae also match the double bond equivalents (DBE) for the proposed structures (B and C). The potential acetylation sites (“R”) are highlighted on the different arabitol C18 congeners (C), as well as the potential number of structural combinations that exist for 3 acetyl groups—the most abundant type of arabitol 3-hydroxy C18.

Consistent with our GC-MS data, we also detected an arabitol acetylation series of 3-hydroxy C18 compounds (Fig 5A), with the structure of the base compound shown in Fig 5B. Similar to mannitol, the 3 acetyl groups was the most abundant version; only arabitol (3 acetyl groups) with 3-methoxy C18 and C18 (one double bond) fatty acids were detected by LC-MS. A mannitol and arabitol acetylation series of 3-hydroxy C16 compounds were also detected, but at a lower abundance compared to the 3-hydroxy C18 compound series (S1 and S2 Figs).

Given these initial findings, we hypothesized that hypoacetylated PEFA compounds (produced by *R*. *taiwanensis*) may be more surface active than those reported for *R*. *babjevae;* less acetylation means more hydroxyl groups to interact with water, making the biosurfactants more hydrophilic. Hyperacetylated species would have a higher surface tension (i.e. the hydroxyl groups are “capped” with the acetyl moiety making the biosurfactants more hydrophobic). Unfortunately, no surface tension measurements were reported by Garay et al. and Cajka et al. on *R*. *babjevae* PEFA compounds [11, 12]. Therefore we characterized a *R*. *babjevae* strain acquired via M.J. Daly (MD1169) through the Ex Culture Collection of Extremophilic Fungi, a part of the Infrastructural Centre Mycosmo (MRICUL) at the Department of Biology, University of Ljubljana, Slovenia.

**Fig 6:**
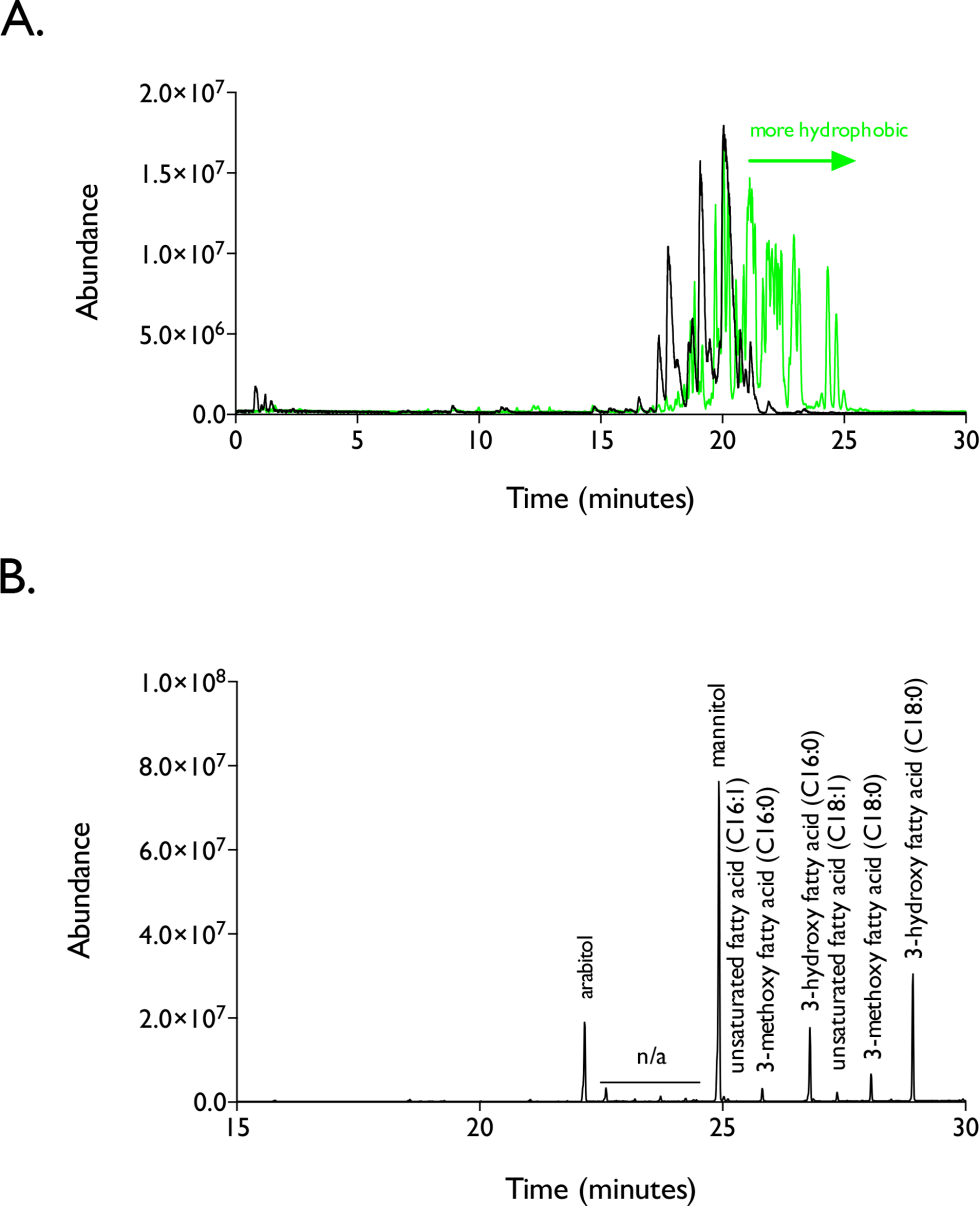
Biosurfactants produced by *R*. *babjevae* MD1169 are polyol fatty acid esters with a similar composition profile to *R*. *taiwanensis* MD1149. *R*. *babjevae* biosurfactants were purified using solid phase extraction (reversed phase), and eluted from the column using 100% methanol as detected by LC-MS analysis (A). *R*. *babjevae* MD1169 compounds are illustrated in the green LC-MS total ion chromatogram, and are overlayed with the LC-MS total ion chromatogram from *R*. *taiwanensis* MD1149 (black trace). Interestingly, GC-MS analysis of *R*. *babjevae* MD1169 revealed that the biosurfactant mixture was composed of the same sugar alcohol and fatty acid constituents as *R*. *taiwanensis*, but at different ratios (B). The GC-MS total ion chromatograms (between the two samples) was normalized for mannitol concentration, and the ratios of the other constituents were relative to it.

LC-MS analysis of spent liquid medium from *R*. *babjevae* MD1169 revealed a series of more hydrophobic compounds than those that were already characterizing for *R*. *taiwanensis* MD1149. This increase in hydrophobicity was detected in the chromatographic profile of the purified compounds as they eluted later in the LC run (at a higher organic phase concentration) which is illustrated in Fig 6A; the elution profile of *R*. *taiwanensis* MD1149 is shown in black, while the elution profile for *R*. *babjevae* MD1169 is shown in green. This shift was consistent with the hypothesis that hyperacetylated forms of PEFA were being produced by *R*. *babjevae* MD1169. When we performed acid digestion, silylation, and GC-MS analysis on this mixture, the composition of the compounds was virtually the same as *R*. *taiwanensis* MD1149, although the relative ratio of the individual components was slightly different (Fig 6B). Note that the GC-MS data for both strains is normalized to mannitol (TMS). Accurate mass LC-MS analyses confirmed that *R*. *babjevae* MD1169 produces essentially the same “base” PEFA compounds, but hyperacetylated congeners compared to *R*. *taiwanensis* MD1149. Mass lists for these compounds are shown in S3 and S4 Figs.

**Fig 7:**
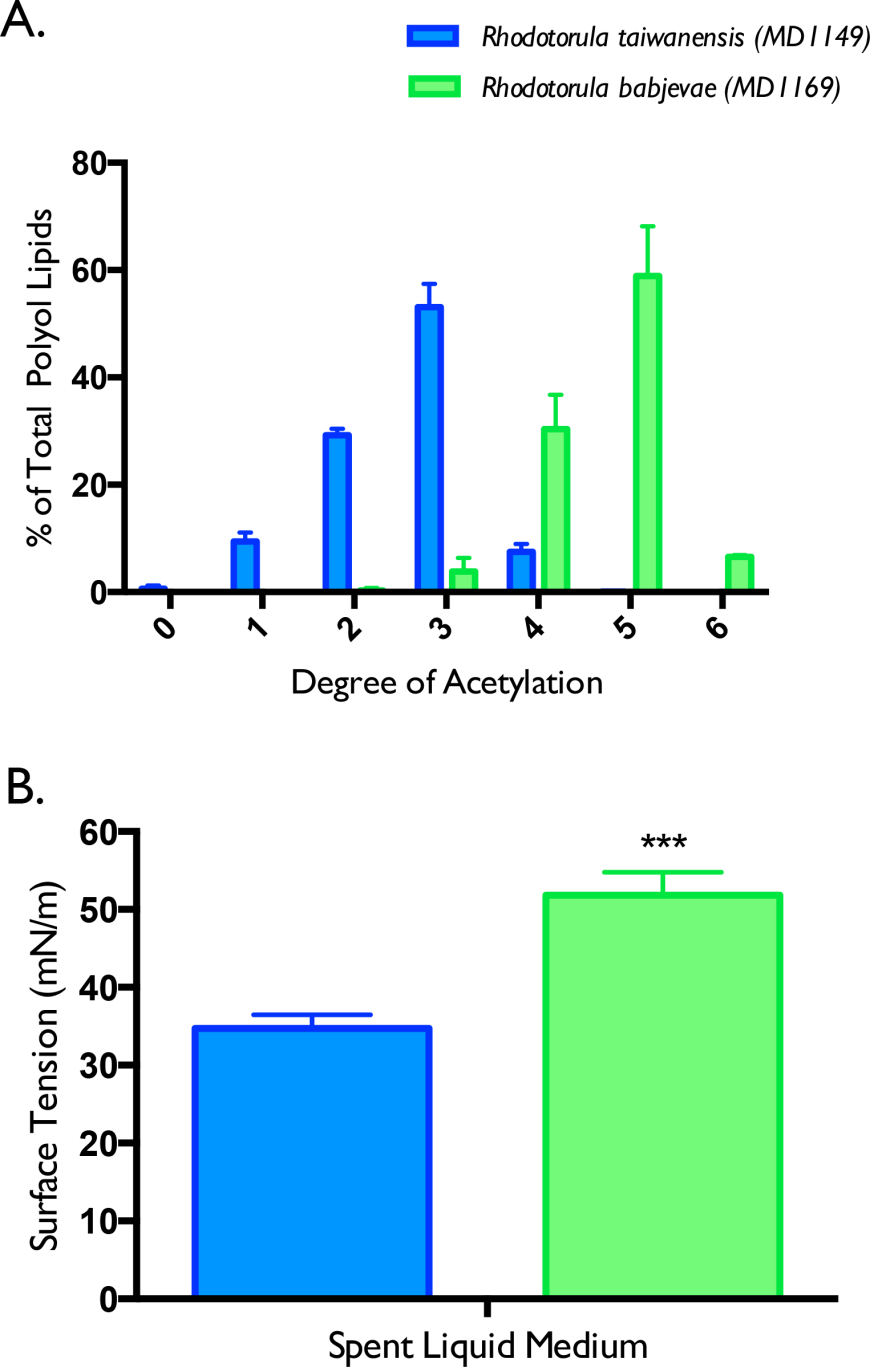
Biosurfactants produced by *R*. *taiwanensis* MD1149 and *R*. *babjevae* MD1169 have distinct acetylation profiles that impact their surface-active properties. Spent liquid medium was harvested during peak production of biosurfactants in three replicate experiments (for each organism), and analyzed by LC-MS. The individual areas for each acetyl group species were then added together to create an acetylation profile of all of the detectable compounds produced by *R*. *taiwanensis* (blue) versus *R*. *babjevae* (green) (A). The surface tension of the spent liquid medium for each of the three biological replicates was also measured, averaged together, and compared between *R*. *taiwanensis* (blue) versus *R*. *babjevae* (green) (B). A p-value less than 0.001 was indicated by the three asterisks.

In order to better represent the acetylation pattern differences between these two strains, we compared the acetylation profiles of the biosurfactants produced in three independent biological replicate cultures for each organism. Spent liquid medium was harvested during peak production of the biosurfactants (prior to biodegradation), and run by LC-MS. The LC-MS data files were then searched in MassHunter using a custom polyol fatty acid database that we constructed from *R*. *taiwanensis* and *R*. *babjevae* data files. The database represented the molecular formula of the polyol fatty acid previously described along with all of the potential acetylation congeners of the base compounds. We deconvolved the mixture by matching the individual compounds using MassHunter software, and measuring the relative abundance of each compound through total area (after peak integration). The compounds were then parsed into the number of acetyl groups they contained, and the relative areas were added together to create an acetylation distribution of all of the detectable compounds. The acetylation distribution is shown in Fig 7A, with a marked Gaussian distribution profile for both strains: *R*. *taiwanensis* peaking with 3-acetyl polyol fatty acid species, and *R*. *babjevae* peaking with 5-acetyl polyol fatty acid species. Without an authentic PEFA standard, it is not possible to determine an absolute concentration of the PEFA compounds in the spent liquid medium by LC-MS. Therefore, we only compared the *relative* abundances of these PEFA compounds between the different strains using peak integrations of the total ion chromatogram (i.e. we integrated areas for each of the compound species in the chromatogram, and added them together for a “total integrated area” per sample). These preliminary comparisons suggested that equal amounts of PEFA were being produced by *R*. *taiwanensis* and *R*. *babjevae*, but with different acetylation profiles. We are currently testing different chemical synthetic routes to produce a PEFA standard for absolute quantitation of these compounds in our spent liquid medium.

To determine if this acetylation profile impacted the surface-active properties of the cultures, we measured the surface tension of the same three biological replicates for *R*. *taiwanensis* MD1149 versus *R*. *babjevae* MD1169. Interestingly, we found that there was a significant difference in surface tension between *R*. *taiwanensis* (blue) and *R*. *babjevae* (green) culture medium: 35 and 52 mN/m, respectively, with a p-value less than 0.001 (Fig 7B). These data were consistent with hypo-acetylated species having a lower surface tension (i.e. more hydroxyl groups to interact with water, biosurfactants more hydrophilic) and hyper-acetylated species having a higher surface tension (i.e. the hydroxyl groups were “capped” with the acetyl moiety making the biosurfactants more hydrophobic). These findings support the notion that acetylation of hydroxyl groups on the same polyol fatty acid esters impacted the hydrophilic-lipophilic (HLB) balance of these compounds.

## Conclusions

We report here the characterization of a unique, hypoacetylated mixture of PEFA compounds which are produced by the environmental isolate *R*. *taiwanensis* MD1149. These compounds are produced within an acetylation range of 0–4 acetyl groups; this range impacts the hydrophilic-lipophilic balance (HLB) of the mixture, making them more surface active than the hyperacetylated PEFA produced by *R*. *babjevae* MD1169, and other recently published PEFA compounds [11].

It is now of keen interest to determine the difference in acetylation patterns between the biosurfactants produced by *R*. *taiwanensis* MD1149 and *R*. *babjevae* MD1169. It is unclear which acetyltransferase(s) mediate the transfer of the acetyl group to the polyol fatty acid ester. Budding yeast encode multiple histone acetyltransferases (HATs)/lysine acetyltransferases (KATs) which use acetyl-CoA as a substrate to transfer acetyl groups to histones and non-histone proteins [25, 26]. It is more probable that the enzyme is a fungal carbohydrate transacetylase similar to the acetyltransferase from *Starmerella bombicola*, which mediates the acetylation of *de novo* synthesized sophorolipid biosurfactants [27]. Interestingly, deletion of this enzyme gene results in the production of only unacetylated sophorolipids, which impacts the physical-chemical properties of these compounds [27] (note: sophorolipids are acetylated at *two* potential positions, versus *six* for mannitol 3-hydroxy C18). The genome of *R*. *taiwanensis* has recently been sequenced, which will allow for a thorough homology comparison of other carbohydrate transacetylases in the NCBI database to *R*. *taiwanensis*; additionally, this may lead to carbohydrate transacetylase identification within the genome of *R*. *babjevae* (a strain of *R*. *babjevae* was sequenced by the DOE Joint Genome Institute under its old designation *Rhodosporidium babjevae*). Measuring the expression levels of this unidentified transacetylase, along with comparing the protein sequence, will likely provide the clues to understanding the acetylation pattern differences between *R*. *taiwanensis* and *R*. *babjevae*.

The general utility of surfactants—either from synthetic or biological origin—is determined by their hydrophilic-lipophilic balance (HLB). The HLB scale was originally proposed by William Griffin in 1949 to measure the degree by which a surfactant is hydrophilic (“water-loving”) or lipophilic (“oil loving”) [28, 29]. The HLB scale ranges from 0–20; surfactants that score >10 are more hydrophilic, and mediate oil-in-water emulsions (e.g. detergents and solubilizers), while those that score <10 are hydrophobic and mediate water-in-oil emulsions (e.g. wetting agents) [30]. This solubility property is a key indicator of a surfactant’s utility within an industrial process. The primary market barrier of biosurfactants is the vast library of existing chemical surfactants. Hundreds of petroleum-derived chemical surfactants cover the entire HLB scale. End users can choose among an array of existing chemical surfactants to identify the best one(s) to meet specific industrial applications. Our findings suggest that acetylation changes the surface chemistry of these PEFA compounds, which directly impact their HLB. Therefore, hypo- and hyperacetylated mixtures of these biosurfactants may have different utilities within the surfactant industry. It will also be important to thoroughly test the solubility of these purified PEFA compounds, and determine the best solvent for each mixture (this may impact the type of industrial application where they are used). Even though these compounds are produced in an aqueous environment, they may not be fully-soluble in water (e.g. hyperacetylated PEFA compounds may lose their surfactant activity, and exist as an oily layer on top of the water). Our preliminary data support this idea, as *R*. *babjevae* produced the same relative amounts of PEFA compared to *R*. *taiwanensis* MD1149 (based on total peak integration area of the LC-MS chromatograms), but had a markedly higher surface tension in the spent liquid medium.

Biosurfactants offer a more environmentally friendly option than chemical surfactants because they are biodegradable and petroleum-independent. However, there are currently less than five commercially-available biosurfactants, and all of them cover only a small portion of the HLB scale. Therefore, they can only be used in very targeted commercial applications. Although environmentally-friendly biosurfactants can reduce our nation’s oil dependence, they will not be broadly used commercially until biosurfactants span more of the HLB scale (i.e. are more useful to disparate industries). Therefore, the finding that PEFA compounds can be produced with a range of acetylation modifications opens the possibility of separating out different species of PEFA compounds with different surface-active properties (i.e. a different HLB value). This is a current focus of our research.

Continuing to identify new biosurfactant species, especially those that fill gaps within the HLB scale, will assist with biosurfactants becoming a viable alternative to chemical surfactants used at the industrial scale. These biosurfactant species can then be produced in large quantities through modern bioengineering techniques, and minimize toxic surfactant waste that impact the environment.

## Supporting information

S1 Fig Mannitol 3-hydroxy C16 exists as an acetylation series. High-resolution mass spectrometry confirmed non-acetylated and acetylated mannitol 3-hydroxy C16 congeners in the spent liquid medium.
(EPS)

S2 Fig Arabitol 3-hydroxy C16 exists as an acetylation series. High-resolution mass spectrometry confirmed non-acetylated and acetylated arabitol 3-hydroxy C16 congeners in the spent liquid medium.
(EPS)

S3 Fig Mannitol fatty acid ester compounds produced by *R*. *babjevae* exist are hyperacetylated. High-resolution mass spectrometry confirmed highly-acetylated mannitol congeners in the spent liquid medium.
(EPS)

S4 Fig Arabitol fatty acid ester compounds produced by *R*. *babjevae* exist are hyperacetylated. High-resolution mass spectrometry confirmed highly-acetylated arabitol congeners in the spent liquid medium.
(EPS)
